# STIM1 accelerates cell senescence in a remodeled microenvironment but enhances the epithelial-to-mesenchymal transition in prostate cancer

**DOI:** 10.1038/srep11754

**Published:** 2015-08-10

**Authors:** Yingxi Xu, Shu Zhang, Haiying Niu, Yujie Ye, Fen Hu, Si Chen, Xuefei Li, Xiaohe Luo, Shan Jiang, Yanhua Liu, Yanan Chen, Junying Li, Rong Xiang, Na Li

**Affiliations:** 1 School of Medicine, Nankai University, 94 Weijin Road, Tianjin 300071, China; 2 Department of Obstetrics and Gynecology, First Central Hospital Clinic Institute, Tianjin Medical University, 24 Fukang Road, Tianjin 300192 China; 3 School of Physics, Nankai University, 94 Weijin Road, Tianjin 300071, China; 4 Beijing Health Vocational College, 94 Nanhengxijie Street, Beijing, 100053 China; 5 Tianjin Key Laboratory of Tumor Microenvironment and Neurovascular Regulation, Tianjin 300071, China; 6 Collaborative Innovation Center for Biotherapy, Nankai University, 94 Weijin Road, Tianjin 300071, China; 7 State Key Lab of Experimental Hematology, Institute of Hematology and Blood Diseases Hospital, Chinese Academy of Medical Sciences, Tianjin 300020, China

## Abstract

The importance of store-operated Ca^2+^ entry (SOCE) and the role of its key molecular regulators, STIM1 and ORAI1, in the development of cancer are emerging. Here, we report an unexpected dual function of SOCE in prostate cancer progression by revealing a decrease in the expression of STIM1 in human hyperplasia and tumor tissues of high histological grade and by demonstrating that STIM1 and ORAI1 inhibit cell growth by arresting the G0/G1 phase and enhancing cell senescence in human prostate cancer cells. In addition, STIM1 and ORAI1 inhibited NF-κB signaling and remodeled the tumor microenvironment by reducing the formation of M2 phenotype macrophages, possibly creating an unfavorable tumor microenvironment and inhibiting cancer development. However, STIM1 also promoted cell migration and the epithelial-to-mesenchymal transition by activating TGF-β, Snail and Wnt/β-Catenin pathways. Thus, our study revealed novel regulatory effects and the mechanisms by which STIM1 affects cell senescence, tumor migration and the tumor microenvironment, revealing that STIM1 has multiple functions in prostate cancer cells.

The concept of store-operated Ca^2+^ entry (SOCE) was first proposed to describe the process whereby the depletion of intracellular Ca^2+^ stores causes the movement of extracellular Ca^2+^ into cells^1^. Recent studies have identified stromal interaction molecule 1 (STIM1) and CRAC modulator 1 (CRACM1, also known as ORAI1) as the key components of SOCE channels^2^,^3^,^4^; these proteins functionally interact with each other to mediate SOCE activity^5^.

Intracellular Ca^2+^ homeostasis is required for many physiological and pathophysiological process, including cell adhesion^6^, secretion^7^, exocytosis^8^, transcription^9^, cell division and cell death^10^,^11^. As a primary regulatory mechanism, SOCE plays a vital role in these processes. Previous studies revealed the overexpression of STIM1 and/or ORAI1 in various types of cells, such as early stage cervical cancer cells^12^ and hepatocellular carcinoma cells^13^. Up-regulation of SOCE has been reported to promote the proliferation in many types of cells, including normal cells, such as endothelial progenitor cells^14^,^15^, human aortic smooth muscle cells (hASMCs)^16^ and human umbilical endothelial cells^17^, as well as tumor cells, such as hepatic cell carcinoma^18^. These results provide evidence that SOCE may play an important role in tumor development, and the targeting of SOCE holds promise as a strategy for suppressing tumorigenesis and tumor proliferation^19^.

Recent studies have also demonstrated that SOCE contributes to migration in various types of cells, including mouse neutrophils^20^, hASMCs and cancer cells etc^6^,^21^. By promoting the entry of extracellular Ca^2+^ to the cytosol, SOCE activates Ca^2+^-dependent proteinases, such as calpain, focal adhesion kinase, and small GTPases, such as Rac, to promote the assembly and disassembly of focal adhesion, thereby accelerating migration^6^,^22^. Blocking SOCE activity by using a specific blocker or by applying siRNAs that target STIM1 and ORAI1 can inhibit the formation of focal adhesions, thus reducing the migration and invasion of tumor cells^6^,^13^. SOCE has also been shown to contribute to angiogenesis by up-regulating the expression of VEGFA^12^ and by affecting the growth and tubulogenesis activity of tumor endothelial progenitor cells^15^. Thus, SOCE contributes to tumor development, suggesting that blocking SOCE activity represents a promising strategy to prevent metastasis.

However, SOCE has also been shown to contribute to apoptosis. Reduced SOCE activity was revealed to be closely correlated with anti-apoptosis properties in prostate cancer cells^23^,^24^. Further studies have shown that that SOCE functionally interacts with the pro-apoptotic protein during apoptosis^25^ and that the overexpression of STIM1 to increase SOCE activity can accelerate apoptosis^26^. In addition, enhanced SOCE signaling hinders tuberous sclerosis complex (TSC)-related tumor growth^27^. Consequently, blocking SOCE activity either by depleting STIM1 or by overexpressing dominant-negative Orai1 can accelerate the development of TSC-related tumors^27^. These findings support the theory that enhancing SOCE activity can be an effective method to increase the sensitivity of tumors to apoptotic stimuli and restrain tumor development.

These conclusions appear different to each other but indicate that SOCE may have distinct effects on regulating tumor progression. To elucidate this hypothesis, the expression levels of STIM1 and ORAI1 were tested in human prostate cancer tissues. Although STIM1 levels were decreased in hyperplasia and tumor patients, this protein was expressed at significantly higher levels in tumors at low histological grade than in hyperplasia tissues. Further studies revealed that the ectopic expression of STIM1 and ORAI1 inhibits tumor cell growth and promotes cell senescence. In addition, STIM1 overexpression significantly promoted the epithelial-to-mesenchymal transition (EMT) and increased the migration of human prostate cancer cell lines in remodeled tumor microenvironments. These results support a dual role of SOCE in human prostate cancer progression and indicate that although targeting of SOCE is a promising strategy for treatment of prostate cancer, the details should depend on the individual situation.

## Materials and Methods

### Ethics statement

All methods including the animal experimentation were carried out in accordance with the approved guidelines of the Institute Research Ethics Committee at Nankai University, and all efforts were made to minimize animal suffering during the experiment.

### Gene cloning

pCDH1-ORAI1-EF1α-puro and pCDH1-STIM1-YFP-EF1α-Bsd vectors were kindly provided by Dr. Xiangdong Tang (School of Medicine, Nankai University). cDNA of YFP was subcloned into the pCDH1-MCS-EF1α-puro vector between the BamHI and XbaI restriction sites.

### Cell culture

Wild-type (Wt) DU145 and U937 cells were purchased from the Cell Resources Center of the Biological Sciences Institute in Shanghai of Chinese Academy of Sciences; BPH-1, PC3 and LNCaP cells were kindly provided by Dr. Ju Zhang (School of Life Science, Nankai University) and cultured as described previously^28^. DU145-Wt and PC3-Wt cells were infected with lentivirus carrying the pCDH1-YFP-EF1α -puro or pCDH1-STIM1-YFP-EF1α-Bsd plasmid, followed by clonal selection using Puromycin or Blasticidin (Bsd, 8 μg/mL for DU145 and 4 μg/ml for PC3) to generate polyclones of DU145 and PC3 cells that stably overexpress YFP and STIM1-YFP (DU145-YFP, DU145-STIM1-YFP, PC3-YFP and PC3-STIM1-YFP); DU145-Wt, DU145-STIM1-YFP, PC3-Wt and PC3-STIM1-YFP cells were infected with lentivirus carrying the pCDH1-ORAI1-EF1α-puro plasmid, followed by clonal selection using Puromycin (2 μg/mL for DU145 and 4 μg/mL for PC3) to generate polyclone cells that stably overexpress ORAI1 (DU145-ORAI1, DU145-ORAI1-STIM1-YFP, PC3-ORAI1 and PC3-ORAI1-STIM1-YFP).

U937 cells were cultured in RPMI-1640 media supplemented with 10% fetal bovine serum (FBS) in the presence of 100 u/mL penicillin and 0.1 mg/mL streptomycin. In the stimulation experiments, the culture supernatant from DU145 or PC3 was added to the culture medium of U937 at a volume ratio of 1:3. U937 cells then were collected after 48 hours (h) of treatment.

### Immunohistochemistry

Immunohistochemical staining was performed on paraffin human prostate tissue arrays (Alenabio Company, Shanxi, China) and on the tumor xenograft tissues of NOD/SCID mice. The expression levels of ORAI1 and STIM1 were detected separately in these tissues using polyclonal rabbit antibodies raised against STIM1 or ORAI1 (ProSci Inc.) at a 1:100 dilution. The expression levels of ORAI1 or STIM1 in the tissue microarray were scored according to the percentage of ORAI1 or STIM1-positive cells in each whole area of prostate tissue and their staining intensity. Specifically, percentages ≤10%, 11%–30%, 31%–49% and ≥50% were scored as 1, 2, 3 and 4, respectively; non-significant brown, slight brown, moderate brown and deep brown staining intensities were scored as 1, 2, 3 and 4, respectively. The two scores were then added, and a score of 1–3 was considered weak, a score of 4–6 was considered moderate, and a score of 7–8 was considered strong.

The expression levels of DcR2 and Vimentin in tumor xenografts were detected separately with polyclonal rabbit antibodies raised against DcR2 and Vimentin (Proteintech Group Inc.) at a 1:100 dilution. Images were recorded using an Olympus BX51 Epi-fluorescent microscope (Olympus Co.).

### Western Blotting

Cell lysates from DU145 and PC3 cell lines were prepared using RIPA buffer in the presence of protease inhibitor cocktails and Phosphatase Inhibitor Cocktails 2 and 3 (Sigma-Aldrich) as described previously^29^. Proteins (40 μg) were loaded onto 5–12% Tris-acrylamide gels and blotted with the following antibodies: anti-STIM1 and ORAI1 (ProSci Inc.), anti-N-cadherin, E-cadherin and Apoptosis I Sampler Kit (BD Biosciences), anti-β-catenin and Wnt-1 (Abcam Inc.), anti-β-actin (Santa Cruz Biotechnology Inc.), anti-cyclin D1, DcR2, and Vimentin (Proteintech Group Inc.), anti-p-Smad3 (Epitomics Inc.), anti-claudin-1, Snail, p-Smad2 and Cell Cycle Regulation Antibody Sampler Kit II (Cell Signaling Technology Inc.), and horseradish peroxidase-conjugated secondary antibodies. The results were visualized using a chemiluminescent HRP substrate kit (Millipore) and analyzed using Image J software (National Institutes of Health, Baltimore, MD).The densitometry results were first normalized with that of β-actin and then compared with the control to obtain relative fold changes. The mean value for each blot was averaged from three independent experiments and indicated at the bottom of the blot.

### Isolation of nuclear proteins

Nuclear proteins were isolated as described before^30^. Proteins (30 μg) were loaded on 5–12% Tris-acrylamide gels and subjected to western blotting, and the proteins were detected using antibody raised against β-catenin (Abcam Inc.); lamin A was used as an equal nuclear protein loading control and was detected using an anti-lamin A antibody (Sigma-Aldrich).

### Living cell growth curve

DU145 or PC3 cells (2 × 10^5^) were seeded evenly in each well of 6-well culture dishes; five regions around the center regions of each dish were selected and dynamically recorded under an objective from 12 h after the seeding. The numbers of cells in each image field were counted and averaged from three independent experiments, each recorded from five image fields.

### β-Galactosidase staining

The cells were cultured in six-well plates until 90% confluence and then fixed and subjected to β-Galactosidase staining using a Senescence β-Galactosidase Staining Kit (KeyGEN Biotech, China) following the manufacturer’s instructions. After staining for 12 h, the cells were imaged under a 40 × objective. The numbers of cells in each image field were averaged from three independent experiments, each recorded from five image fields.

### NF-κB activity assay

A Dual-Luciferase Reporter Assay System (Promega) was used to determine the activity of firefly luciferase (FL) versus that of renilla luciferase (RL). Briefly, cells were cultured in 24-well plates at a density of 2 × 10^5^/well and transfected with a DNA mixture containing 300 ng of pGL4.32-Luc2-NFKB-RE plasmid (a kind gift from Dr. Tsung-Hsien Chuang, National Health Research Center, Taiwan) and 30 ng of pRL-TK plasmid. The cells were harvested after transfection for 30 h, and the NFκB-RE activation was quantified as the ratio of FL/RL activity in each well following the manufacturer’s instructions.

### Intracellular Ca^2+^ measurement

Fura-2 loaded prostate cell lines were placed in a balanced salt solution including 0 Ca^2+^ and 0.5 mM EGTA. Intracellular Ca^2+^ was first depleted by applying 1 μM Thapsigargin (TG, Sigma-Aldrich) to the extracellular solution to deplete intracellular Ca^2+^ store thus trigger the opening of SOCE channels and activation of SOCE; then, CaCl_2_ was added to the extracellular solution to a final concentration of 2.5 mM after 300 seconds of TG treatment. The amount of Ca^2+^ entering into the cytosol through the SOCE channels was reflected by an increase in the fluorescent intensity ratio of fura-2 in each cell monitored at 340 nm and 380 nm. All measurements shown are averages measured for 13–67 cells from a minimum of two independent experiments.

### Flow cytometry analysis of the cell cycle

After culturing DU145 and PC3 cells in the absence of FBS for 12 h, the cells were ‘pulsed’ with 10 μM 5-bromo-2-deoxyuridine (BrdU, Sigma-Aldrich) for 24 h at 37 °C, and a cell cycle assay was performed using BD Pharmingen^TM^BrdU Flow Kits (BD Biosciences) following the manufacturer’s instructions.

### Real- time RT-PCR

Total mRNAs from U937, DU145 and PC3 cells were isolated using TRIzol reagent (Invitrogen Inc.) and reverse-transcribed into cDNAs using MMLV reverse transcriptase (Promega). Following this, Real-time RT-PCR was performed on an Opticon instrument (Bio-Rad) in 20-μl reaction volumes using TransStart Green qPCR Super Mix Kit (TransGen Biotech, Beijing, China). Homo GAPDH was used as the internal control. The 2^−ΔΔCt^ method was used to determine fold-changes in the levels of mRNA expression. The results were statistically averaged from three independent experiments that were performed in triplicate. The primers used for the experiments are summarized in Table 1. The primers used for detection of *STIM1* mRNA were described before^28^.

### Wound healing assay

Cells (2 × 10^5^) were seeded in each well of a 24-well plate. At full confluence, a “wound” was made in the middle of culture plate using a 10-μl pipette tip, and the concentration of serum in the tumor cell culture medium was changed from 10% to 1% to avoid the influence of cell growth rate on wound healing. Wound healing was recorded at 0 and 24 or 36 h after scratching under a 10 × objective. The rate of healing was quantified as the distance of wound recovered versus that of original wound, as described before^29^.

### Transwell assay

Transwell chambers (polycarbonate filters with 8 μm porosity, Millipore) were used in the test. The bottom chamber was filled with culture medium containing 10% FBS for the transwell assay of DU145 and PC3. For the transwell assay of U937, the bottom chamber was filled with culture supernatant from DU145 or PC3 cells. Cells (10^5^) were suspended in serum-free medium and plated in the upper chamber. After incubation for certain time courses (24 h for DU145 and PC3 and 12 h for U937), the cells were removed from the upper chamber. Cells that had penetrated and attached to the bottom of the filter were fixed with 4% formaldehyde in PBS and then stained using 0.5% crystal violet; the cells were then imaged under a 20 × objective. The crystal violet was dissolved using 50% acetic acid, and the staining intensity was recorded as the absorbance measured at 560 nm.

### Cytokine array

DU145-YFP and DU145-STIM1-YFP cells (2 × 10^5^) were cultured in six-well plates. After 48 h, 1 mL of supernatant was harvested from each cell line and then subjected to a cytokine array using the Human Cytokine Array Kit (R&D Systems) following the manufacturer’s instructions. Quantitative expression levels of each protein were analyzed using ImageJ software and normalized as described previously^31^. The results were averaged from two independent experiments, which were performed in duplicate pots.

### Tumor xenografts

Male NOD/SCID mice at 8–10 weeks of age were separated randomly into four groups. DU145 cells (DU145-YFP, DU145-STIM1-YFP, DU145-ORAI1, and DU145-ORAI1-STIM1-YFP) (3 × 10^6^) were inoculated subcutaneously into each mouse at the right axilla. The tumors were measured using calipers, and tumor volume (mm^3^) was calculated using the standard formula: length × width^2^/2. Each mouse was intraperitoneally injected with 1mg BrdU 24 h before death.

### Immunofluorescent staining

The expression of E-cadherin in tumor xenografts was detected by using immuneofluorescent staining method as described before^29^.

### Statistical Analysis

**Figure 1:**
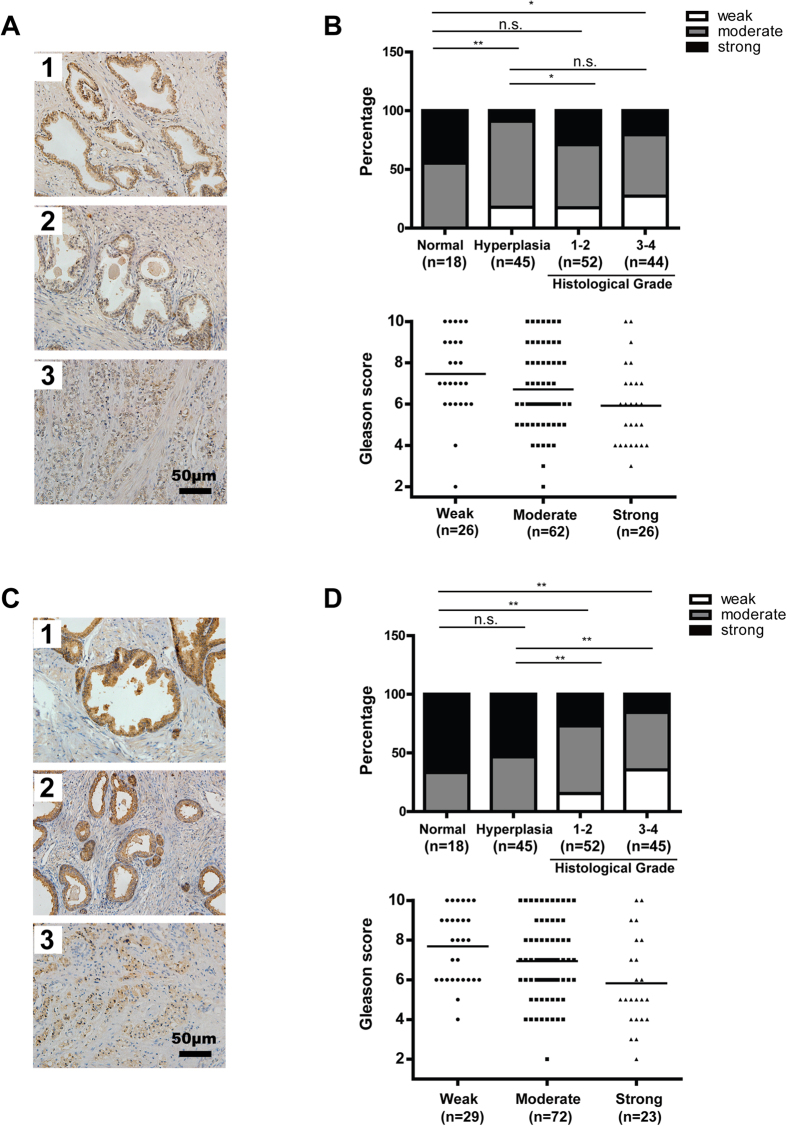
Decreased expression of STIM1 and ORAI1 in human prostate cancer tissues when compared with normal tissues. **A**. Representative immunohistochemical staining of STIM1 (brown) in normal prostate tissue (1), hyperplasia tissue (2) and prostate tumor tissue with a Gleason score of 10 (3). **B.** Upper panel: The expression pattern of STIM1 in normal, hyperplasia and prostate tumor tissues with different histological grades. Low panel: The Gleason score of human prostate cancer tissues with different expression levels of STIM1. The mean value of Gleason score in each group was indicated by horizontal line. **C.** Representative immunohistochemistry staining of ORAI1 (brown) in normal prostate tissue (1), hyperplasia tissue (2) and prostate tumor tissues with a Gleason score of 10 (3). **D.** Upper panel: The expression pattern of ORAI1 in normal, hyperplasia and prostate tumor tissues with different histological grades. Low panel: The Gleason score of human prostate cancer tissues with different expression levels of ORAI1. The mean value of Gleason score in each group was indicated by horizontal line.

Values are expressed as the mean + SEM. The significance of the results shown in Fig. 1 was determined according to the χ^2^ test, and the significance of other results was determined according to *t*-test. A value of *p* < 0.05 was used as the criterion for statistical significance. *indicates significant difference with *p* < 0.05, **indicates significant difference with *p* < 0.01, and “n.s.” indicates no significance.

## Results

### Decreased expression of STIM1 and ORAI1 in human prostate tumor tissues

We first performed an immunohistochemistry analysis of STIM1 and ORAI1 in prostate tissues removed from patients with or without adenocarcinoma cancer (Fig. 1A,C). The expression of STIM1 and ORAI1 in prostate tissues was summarized. As shown in Fig. 1B, STIM1 was detected to be expressed at significantly lower levels in hyperplasia and tumor tissues at histological grade 3–4 than in normal tissues, indicating that these molecules might have an inhibitory role in human prostate tumorigenesis. However, the expression of STIM1 was higher in tumors of histological grade 1–2 than in hyperplasia tissues (Fig. 1B), indicating the possible contribution of STIM1 to the malignant transformation of prostate cells. We also noticed that the averaged Gleason score reduced when STIM1 was expressed at higher levels in prostate cancer tissues (Fig. 1B), suggesting the inhibitory role of STIM1 to development of advanced tumor. These data imply that STIM1 might play a dual role in prostate cancer progression. At the same time, the expression of ORAI1 reduced in prostate cancer when compared with normal and hyperplasia tissues and when the cancer progressed from low to high Gleason scores (Fig. 1D), indicating the prohibitive effect of ORAI1 on development of prostate cancer.

### Human prostate cancer cell lines exhibit higher SOCE activity and STIM1 expression than hyperplasia cells

**Figure 2:**
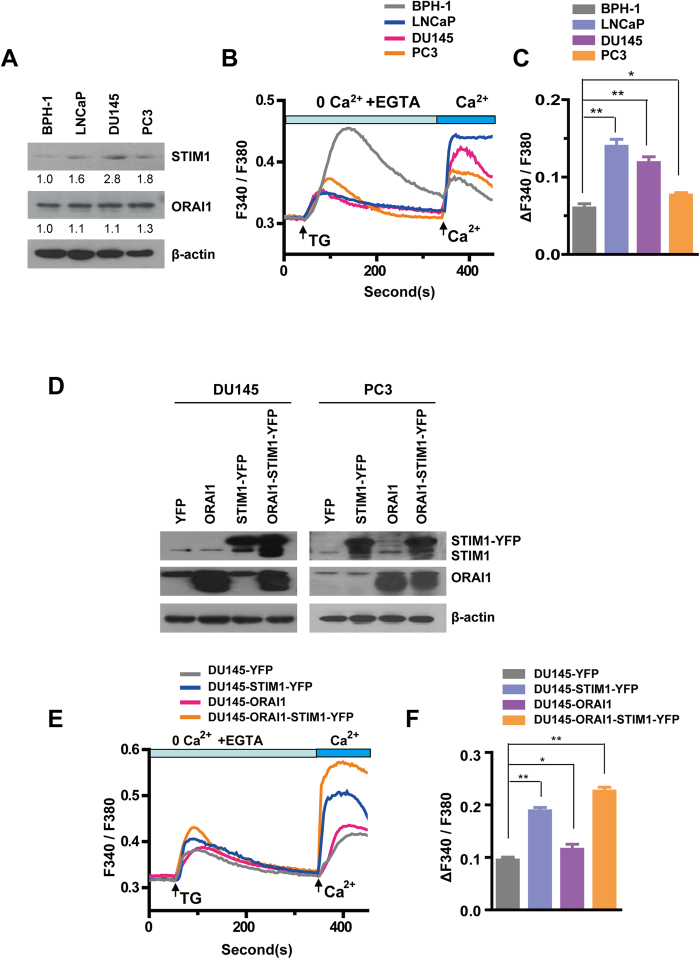
Increased expression levels of STIM1 and SOCE activity in human prostate cancer cells when compared with hyperplasia cells. **A**. Western blotting analysis of STIM1 and ORAI1 in BPH-1, LNCaP, DU145 and PC3 cells. **B**. Comparison of SOCE activity in BPH-1, LNCaP, DU145 and PC3 cells. **C.** Statistical results of SOCE activities in human prostate cancer cells, represented as increases in the F_340_/F_380_ ratio over baseline values (ΔF_340_/F_380_) after extracellular Ca^2+^ application. **D.** Western blotting was used to confirm overexpression of *STIM1* and/or *ORAI1* genes in DU145 and PC3 cells. **E**. SOCE activities in DU145 cells with STIM1-YFP and /or ORAI1 overexpression and the DU145-YFP control. **F.** Increased SOCE activity in DU145-STIM1-YFP and DU145 -ORAI1-STIM1-YFP cells and slightly increased SOCE activity in DU145-ORAI1 cells when compared with that of DU145-YFP control.

Having identified the expression properties of STIM1 and ORAI1 in human prostate tumor tissues, we next examined their expression level in four human prostate cell lines, i.e., BPH-1, LNCaP, DU145 and PC3; BPH-1 is a hyperplasia cell line, and the latter three are cancer cell lines with increasing propensity for metastasis^32^. We found that STIM1 expression is higher in the three prostate cancer cell lines than in BPH-1 (Fig. 2A), suggesting that STIM1 may play a significant role in the development of human prostate cancer. Consistent with our observation in prostate tumor tissues with different Gleason scores, we also noticed that STIM1 expression was significantly lower in the more malignant cell line-PC3 than in the DU145 cells. Because STIM1 is an important component of SOCE channels, we measured cellular SOCE activity using an InCyt dual-wavelength fluorescence imaging system. Although TG, a sarco/endoplasmic reticulum Ca^2+^ ATPase (SERCA) inhibitor, induced endoplasmic reticulum (ER) Ca^2+^ store release was lower (Fig. S1A), SOCE activity was significantly greater in the prostate cancer cell lines than in the BPH-1 cells (Fig. 2B,C), which is consistent with the expression of STIM1 in these cell lines. SOCE activity was also lower in PC3 than in LNCaP and DU145 cells, indicating that STIM1 has dynamic functions during prostate cancer progression.

### Overexpression of STIM1 and ORAI1 in DU145 and PC3 cells inhibits cell growth by arresting the cell cycle in the G0/G1 phase and decreasing the percentage of cells in the G2/M phase

**Figure 3:**
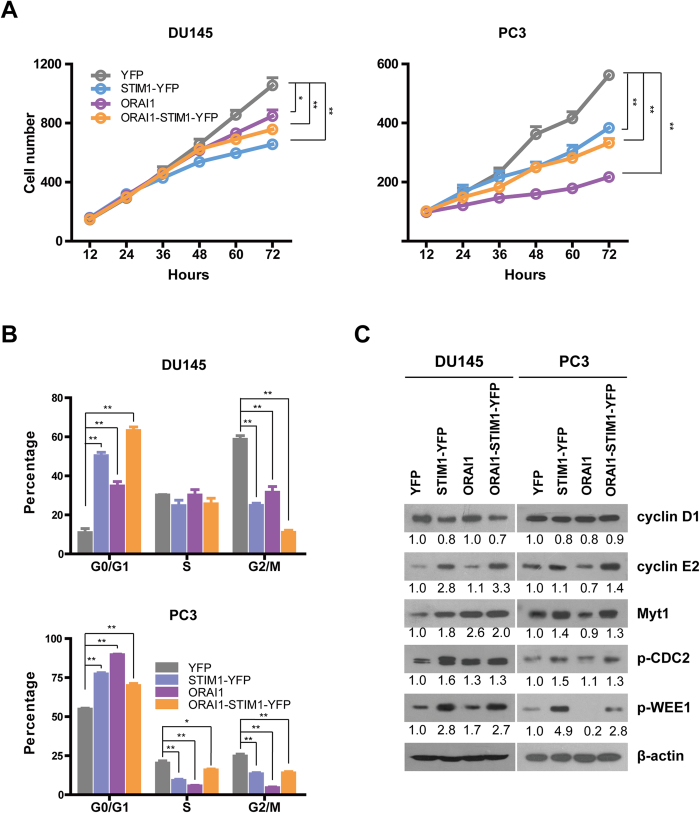
Overexpression of STIM1 and/or ORAI1 slows cell growth and the cell cycle. A. Cell growth curves of DU145 and PC3 cells, n = 3. **B**. The overexpression of STIM1 and/or ORAI1 leads to an increased percentage of cells in the G0/G1 phase and a decreased percentage of cells in the G2/M phase in DU145 and PC3 cells, n = 3. **C**. Western blotting analysis of cell cycle-related proteins in DU145 and PC3 cells upon STIM1 and/ or ORAI1 overexpression.

To further investigate the role of STIM1 and ORAI1 in prostate cancer, we established DU145 and PC3 cell lines that stably overexpressed STIM1-YFP and/or ORAI1. Cells in which YFP was stably expressed (DU145-YFP or PC3-YFP) were used as corresponding controls. STIM1-YFP or/ and ORAI1 overexpression was confirmed by western blotting (Fig. 2D). As shown in Fig. 2E,F, overexpression of STIM1-YFP alone significantly increased SOCE activity in DU145 cells, and this activity could be further enhanced by the co-expression of STIM1-YFP and ORAI1.The same phenomenon was also detected in PC3 cells (Fig.S1B and S1C). Next, we studied the growth of DU145 cells using a live cell growth curve assay. As shown in Fig. 3A, we observed a low growth rate in DU145-ORAI1, DU145-STIM-YFP and DU145-Orai-STIM1-YFP cells, indicating that STIM1 and ORAI1 have inhibitory effects on cell proliferation. A similar phenomenon was also observed in PC3 cells. To further reveal the function of these molecules in cell cycle regulation, DU145 and PC3 cells were serum starved for 12 h and then subjected to cell cycle analysis. We found that DU145 and PC3 cells overexpressing STIM1-YFP, ORAI1 and ORAI1-STIM1-YFP were enriched in the G0/G1 phase and that the percentage of these cells in the G2/M phase was significantly reduced (Fig. 3B and Fig. S2). In addition, the percentage of these PC3 cells in the S phase was also reduced (Fig. 3B and Fig. S2). Western blotting further revealed the overexpression of cyclin E2, which accelerates the G1 phase^33^, and the down-regulation of cyclin D1, which promotes the G1/S transition^34^, in DU145 cells with STIM1-YFP and ORAI1-STIM1-YFP overexpression. In addition, the expression levels of p-WEE1 (Ser642) and Myt1 (inhibitors of cyclin-dependent kinase 1 (Cdk1, also known as CDC2^35^,^36^)) and p-CDC2 (Tyr15) (the inactivated form of Cdk1) were higher in DU145 cells in which STIM1-YFP, ORAI1 and ORAI1-STIM1-YFP were overexpressed than in DU145-YFP control cells; this finding reveals the possible molecular mechanisms underlying the inhibition of cell growth rates in these three cell lines (Fig. 3C). Increased expression of cyclin E2, p-WEE1, p-CDC2 and Myt1 was also detected in PC3 cells in which STIM1-YFP and ORAI1-STIM1-YFP were overexpressed, and a reduction in the levels of cyclin D1 was detected in PC3 cells in which STIM1-YFP, ORAI1 and ORAI1-STIM1-YFP were overexpressed. In addition, the expression level of cyclin E2 was reduced in PC3-ORAI1 cells, possibly accounting for the lower growth rate of PC3-ORAI1 compared to those of PC3-STIM1-YFP and PC3-ORAI1-STIM1-YFP (Fig. 3A).

### STIM1 and ORAI1 promote the senescence of prostate cancer cells

**Figure 4:**
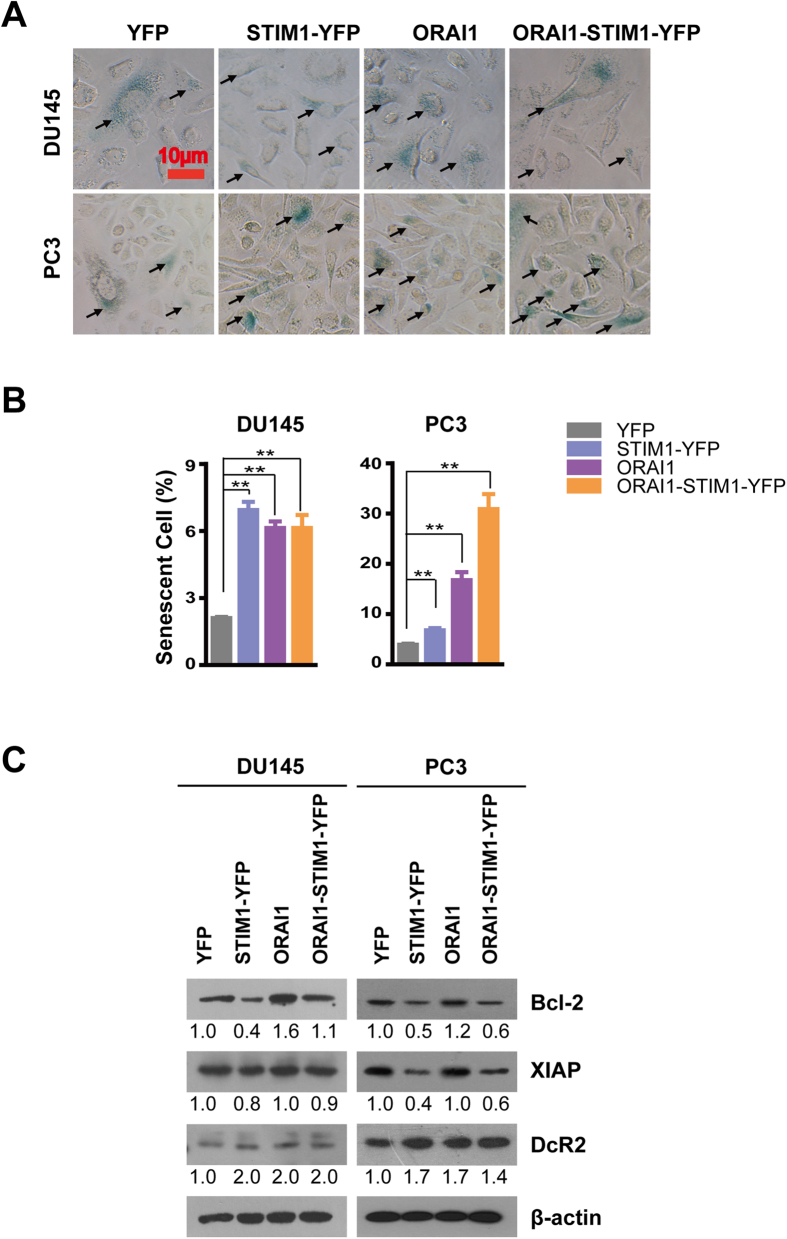
Overexpression of STIM1 and/or ORAI1 promotes cell senescence. **A**. β-Gal staining of senescent cells in DU145 and PC3 cells, the positive cell was indicated by the arrow. **B**. Statistical analysis of the percentage of β-Gal staining positive cells (senescent cells) from DU145 and PC3 in each image field (n = 3). **C**. Western blotting analysis of senescence marker and apoptosis-related proteins in DU145 and PC3 cells.

In addition to the slowed cell growth, we also observed large and long spindle shaped cells with low cell density at full confluence; these characteristics of cell senescence were observed in the DU145-STIM1-YFP, PC3-STIM1-YFP and PC3-ORAI1 cells (Fig. S3A). All these data suggest that the overexpression of STIM1 and/or ORAI1 may promote cell senescence in prostate cancer cells. To further test this hypothesis, we performed β-Gal staining (Fig. 4A) and examined several cell senescent and apoptotic biomarkers (Fig. 4C). As shown in Fig. 4B, the percentages of β-Gal staining (+) cells (senescent cells) were significantly higher in DU145 and PC3 cells overexpressing STIM1-YFP, ORAI1 and ORAI1-STIM1-YFP than in the corresponding controls. In addition, western blotting revealed elevated protein expression levels of DcR2 in DU145 and PC3 cells overexpressing STIM1-YFP, ORAI1 and ORAI1-STIM1-YFP and revealed less expression of anti-apoptotic proteins-X-linked inhibitor of apoptosis protein (XIAP) in DU145 and PC3 cells overexpressing STIM1-YFP and ORAI1-STIM1-YFP. Moreover, the expression of Bcl-2 was much lower in DU145 and PC3 cells overexpressing STIM-YFP and in PC3-ORAI1-STIM1-YFP cells (Fig. 4C). These results revealed possible mechanisms underlying the promotion of senescence by STIM1 and ORAI1 in prostate cancer cells. Since STIM1 contributes significantly to the SOCE activity and overexpression of STIM1 was reported to activate other non-SOCE pathways^37^, to further confirm the regulatory effect of SOCE on cell senescence, we knocked down its expression by applying siRNA mixture to minimize the off-target effect of siSTIM1^38^ (Table S1 and Fig. S3B). Reduced expression of DcR2 and increased Bcl-2 and XIAP were observed in both DU145 and PC3 cells with STIM1 down-regulation (Fig. S3C). In addition, less senescence cells were detected in DU145 and PC3 cells with STIM1 knocking down when compared with their controls (Fig. S3D). These finding further confirm the contribution of SOCE on cell senescence in human prostate cancer cells.

### STIM1 enhances migration by promoting EMT upon the activation of the Snail, TGF-β and Wnt/β-Catenin signal pathways

**Figure 5:**
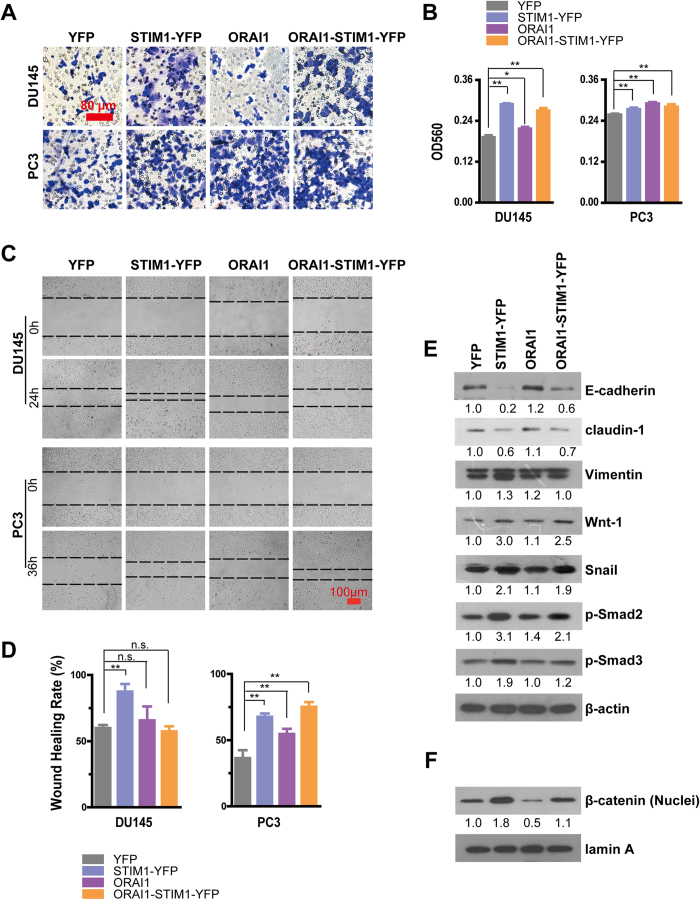
Overexpression of STIM1 and/or ORAI1 promotes the migration of DU145 and PC3 cells. **A.** Representative crystal violet staining for DU145 and PC3 cells that migrated and attached to the bottom of transwell filters after 24 h of treatment. **B**. Statistical analysis of crystal violet staining intensity measured as the absorbance at 560 nm, (n = 3 for DU145 cells, n = 5 for PC3 cells). **C**. Wound-healing assays of cell motility in DU145 and PC3 cells, and representative images obtained at 0 and 24 or 36 h after scratching. The dashed line indicated the edge of the wound. **D.** Statistical analysis of the wound-healing rates of DU145 and PC3 cells, (n = 5 for DU145 cells, n = 3 for PC3 cells). **E**. Western blotting analysis of E-cadherin, claudin-1, Vimentin, Wnt-1, Snail, p-Smad2 and p-Smad3 in DU145 cells. **F.** Western blotting analysis of nuclear β-catenin in DU145 cells.

Because STIM1 and/or ORAI1 overexpression significantly altered the morphology of the DU145 and PC3 cells, we next tested the migration of these cells using transwell (Fig. 5A,B) and wound healing (Fig. 5C,D) assays. As shown in Fig. 5A,B, overexpression of STIM1 alone and/or ORAI1 significantly promoted cell migration in DU145 and PC3 cells. In addition, STIM1 increased the motility of DU145 and PC3 cells in the wound-healing assay, and ORAI1 alone or in combination with STIM1 also significantly promoted PC3 mobility (Fig. 5C,D). EMT is an important cell program in which polarized and immotile epithelial cells lose their polarity and tight cell-cell contacts, and acquire motile mesenchymal characteristics^39^, as marked by the decreased expression of epithelial proteins, such as E-cadherin and claudins and by the increased expression of mesenchymal marker proteins, such as N-cadherin, Vimentin, α-smooth muscle actin and fibronectin etc. Our results showed clearly reduced claudin-1 and/or E-cadherin expression in DU145 and PC3 cell overexpressing STIM1-YFP and ORAI1-STIM1-YFP cells and increased expression of Vimentin or/and N-cadherin in DU145-STIM1-YFP and PC3 cells with STIM1-YFP and ORAI1-STIM1-YFP overexpression (Fig.5E, Fig. S4A and S4B), indicating that STIM1 promotes EMT. However, the effect of ORAI1 on EMT was not significant and was inconsistent between DU145 and PC3 cells because the expression level of E-cadherin, claudin-1 and Vimentin were all slightly increased in DU145-ORAI1 cells, whereas the expression of claudin-1 did not change, the expression of Vimentin increased, but that of N-cadherin decreased in PC3-ORAI1 cells (Fig. 5E and S4A). These results further demonstrated the important function of STIM1 in EMT and its contribution to tumor migration. The regulatory effect of STIM1 on EMT was also confirmed in DU145 and PC3 cells with STIM1 down-regulation as reflected by increased claudin-1, E-cadherin and decreased Vimentin expression in DU145-siSTIM1 cells and elevated claudin-1 and reduced N-cadherin and Vimentin expression in PC3-siSTIM1 (Fig. S4C and S4D).To dig deeper into the underlying mechanism, EMT regulatory proteins were detected in DU145 and PC3 cells by western blotting (Fig. 5E,F, Fig. S4A). Interestingly, Snail, Wnt-1, nuclear β-catenin and p-Smad2, p-Smad3, which are the downstream molecules of TGF-β signaling^40^ were all overexpressed in DU145-STIM1-YFP and DU145-ORAI1-STIM1-YFP cells, whereas the expression of p-Smad2 increased, but that of Wnt-1, Snail only marginally increased and the expression of nuclear β-catenin decreased in DU145-ORAI1 cells. At the same time, β-catenin, Wnt-1, Snail, p-Smad2 and p-Smad3 overexpression was also observed in PC3-STIM1-YFP and PC3-ORAI1-STIM1-YFP cells. However, although p-Smad3 was overexpressed, the expression of Snail and p-Smad2 only slightly increased and that of β-catenin and Wnt-1 did not change in PC3-ORAI1 cells. These results suggest that STIM1 contributes more significantly to EMT than ORAI1 in human prostate cancer cells.

### STIM1 and ORAI1 modulate the tumor microenvironment (TME) and inhibit the formation of tumor-associated macrophages

**Figure 6:**
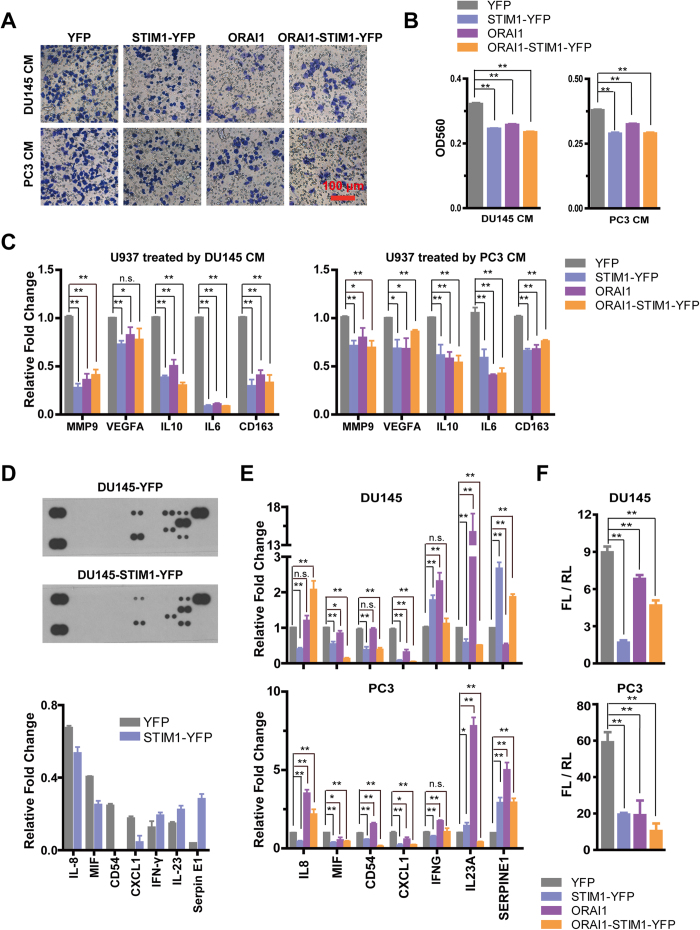
Overexpression of STIM1 and/or ORAI1 in DU145 and PC3 cells remodels the tumor microenvironment. **A.** Transwell assay of the migration of U937 cells after incubation with the conditioned medium (CM) from DU145 and PC3 cells in the low chamber for 12 h; representative crystal violet staining of U937 cells that migrated and attached to the bottom of the transwell filter are shown. **B.** Statistical analysis of violet staining intensity, measured as the absorbance at 560 nm (n = 3). **C.** Real-time RT-PCR analysis of mRNA expression changes of *MMP9*, *VEGFA*, *IL10*, *IL6* and *CD163* in U937 cells after treatment with the CM of DU145 and PC3 for 48 h (n = 3). **D.** STIM1 regulates the expression of cytokines in DU145 cells. Upper panel: representative results of cytokine arrays for DU145-YFP and DU145-STIM1-YFP. Lower panel: Statistical analysis of the relevant protein expression level of cytokines in DU145 cells. **E.** Real-time RT-PCR analysis of mRNA expression changes of *IL8*, *MIF*, *CD54*, *CXCL1*, *IFNG*, *IL23A* and *SERPINE1* in DU145 and PC3 cells with STIM1-YFP and /or ORAI1 overexpression in comparison with those in control cells. **F.** Dual luciferase assay for NF-κB-RE activation in DU145 and PC3 cells.

Stroma cells from the TME are essential for the growth, chemoresistance and metastasis of cancer cells; the transition of macrophages from the M1 to the M2 phenotype is a vital factor in modulating the TME, thus promoting tumorigenesis and metastasis^41^. To elucidate the function of SOCE in modulating the TME and tumor-associated macrophages (TAM, M2 phenotype), we tested the effect of STIM1 and ORAI1 on recruitment U937, a human leukemic monocyte macrophage cell line, in both DU145 and PC3 cells. Our study found that the recruitment of U937 is inhibited by the conditioned medium (CM) from DU145 and PC3 cells overexpressing either *STIM1-YFP* or *ORAI1* or both genes (Fig. 6A,B) and increased recruitment of U937 was demonstrated by using the CM from DU145-siSTIM1 and PC3-siSTIM1 when compared with the control from transwell assay (Fig.S5). In addition, real time RT-PCR showed reduced expression levels of *IL10*, *IL6* and *CD163* in U937 cells after treatment with CM from DU145 and PC3 cells overexpressing STIM1-YFP and /or ORAI1 (Fig. 6C), indicating that U937 cells experienced a transition from the M2 to the M1 phenotype and that the recruitment of these macrophages was inhibited by the TME of prostate cells possessing enhanced SOCE activity. In addition, the transcripts of *VEGFA* and *MMP9* in U937 were also significantly reduced after treatment with the CM from DU145 or PC3 cells overexpressing STIM1-YFP and /or ORAI1, suggesting that the TME of prostate cancer cells with enhanced SOCE activity may be unfavorable to tumor growth and metastasis. To test this hypothesis, we studied the possible effect of STIM1 on regulating the secretion of cytokines in prostate cancer cells using the cytokine array. The results showed that STIM1 overexpression reduced the secretion of IL-8, macrophage migration inhibitory factor (MIF), CD54 (also known as intercellular adhesion molecule 1), and CXC ligand 1 (CXCL1) and increased the secretion of IFN-γ, IL-23 and Serpin E1 in DU145 cells (Fig. 6D). The screening results were further verified by real time RT-PCR in both DU145 and PC3 cells overexpressing STIM1 (Fig. 6E), except that the mRNA expression of *IL23A* decreased in DU145-STIM1-YFP and that of *IFNG* reduced in PC3-STIM1-YFP. It is reported that CXCL1 boosts tumor angiogenesis and development^42^. IL-8 contributes to tumor progression and metastasis^43^. MIF regulates innate immunity^44^ and promotes the metastasis of colorectal cancer^45^. CD54 promotes prostate tumor metastasis^46^. IL-23 enhances the production of IFN-γ^47^. Serpin E1, a serine protease inhibitor, functions as the principal inhibitor of urokinase (uPA) and tissue plasminogen activator (tPA), thus playing an important role in regulating fibrinolysis. With the exceptions of the no significant change for the mRNA expression of *IL8* and *CD54* in DU145-ORAI1 and increase expression of these two genes in PC3 cells with ORAI1 overexpression and the decrease in the mRNA expression of *SERPINE1* in DU145-ORAI1 cells, the effects of ORAI1 on regulating the expression of these cytokine genes are similar to the effects of STIM1 on the secretion of the corresponding proteins observed in DU145 cells (Fig. 6E). These findings support the hypothesis that STIM1 and ORAI1 overexpression promote the formation of an unfavorable TME, which inhibits the development of advanced prostate cancer. In addition, a dual luciferase assay revealed that STIM1 and/or ORAI1 overexpression significantly inhibited the activation of response element (RE) for NFκB (NFκB-RE) (Fig. 6F); this might represent the mechanism that alters the production of cytokines as observed in both the DU145 and PC3 cells.

### STIM1 regulates tumor cell proliferation and cell senescence in *vivo*

**Figure 7 f7:**
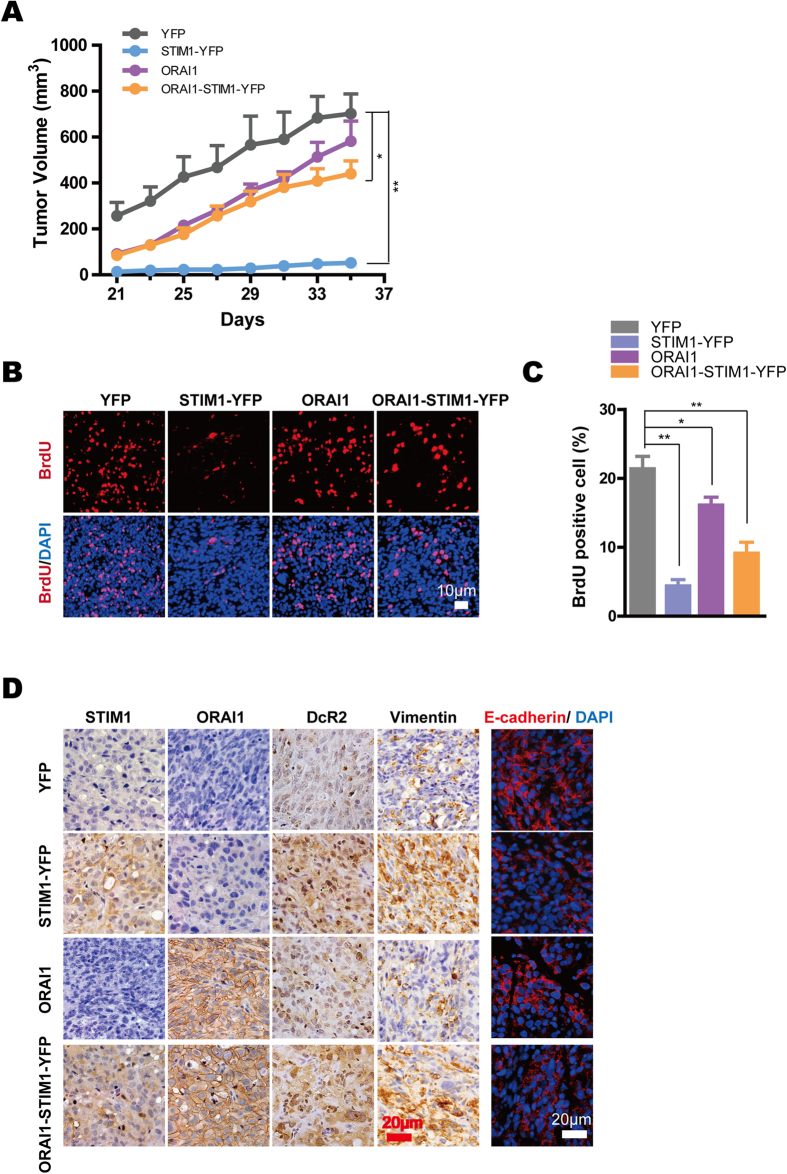
STIM1 in DU145 cells compromise tumor growth with acceleration of the EMT and increased cell senescence in tumor xenografts in *vivo*. **A.** Tumor growth curves in NOD/SCID mice inoculated with DU145-YFP (n = 5), DU145-STIM1-YFP (n = 6), DU145-ORAI1 (n = 4), and DU145-ORAI1-STIM1-YFP (n = 4). **B.** Immunofluorescent image of BrdU in tumor xenograft tissues; the images represent one of 4–6 mice from each group. **C.** Statistical analysis of BrdU-positive cell percentage from tumor xenograft tissues in each image field. **D.** Immunohistochemical staining of STIM1, ORAI1, DcR2 and Vimentin (all in brown) and immunofluorescence staining of E-cadherin (red) in different tumor xenograft tissues.

Next, we studied the roles of STIM1 and ORAI1 in prostate tumor progression in *vivo*. DU145 cells were inoculated into non-obese diabetic-severe combined immunodeficient (NOD/SCID) mice, and noticeable tumor masses were detected within 3 weeks of inoculation. As shown in Fig. 7A, although the tumor volume steadily increased in all four xenograft groups, tumor growth was significantly retarded in mice injected with DU145-STIM1-YFP or DU145-ORAI1-STIM1-YFP compared with that in mice injected with DU145-YFP. The mice were killed 35 days after inoculation. Significantly reduced immunofluorescence staining of BrdU positive cells (Fig. 7B[Fig f7]C), and increased Immunohistochemical stainings of DcR2 were found in DU145-STIM1-YFP, DU145-ORAI1 and DU145-STIM1-YFP-ORAI1 tumor xenografts (Fig. 7D); these findings support the idea that STIM1 and ORAI1 inhibit the tumor cell cycle and accelerate senescence. In addition, immunofluorescent staining of E-cadherin was decreased in tumor tissues that were removed from mice grafted with DU145-STIM1-YFP and DU145-STIM1-YFP-ORAI1, further demonstrating that STIM1 promotes the EMT in *vivo* (Fig. 7D).

## Discussion

In this study, we explored the role of STIM1 and ORAI1 in tumor senescence, migration and microenvironment and demonstrated their dual functions during prostate cancer progression. Our data showed that the two genes can both accelerate cell senescence and promote an unfavorable TME and promote tumor migration and further revealed the molecular mechanisms underlying the effect of STIM1 on EMT regulation in human prostate cancer cells.

Previous study investigated the mRNA expression of *STIM1* and *ORAI1* in prostate tissues by using qRT-PCR and found no significant difference between normal and cancer tissues^48^, which was different with our immunohistochemistry results. These findings indicate the possible post-translational modification for these two genes in prostate cancer cells.

Other isoforms of STIM and ORAI, such as STIM2, ORAI2 and ORAI3 and some transient receptor potential (TRP) family members that involved in the regulation of SOCE activity might also be differently expressed in prostate cancer cells, which may account for the phenomenon observed in Fig. 2A that although DU145 and PC3 showed more STIM1 expression and almost similar or slight increase of ORAI1 when compared with those of LNCaP, their SOCE activity obviously decreased. In addition, since different cancer cell lines have distinct gene background, other genes besides STIM1 that also impact EMT process may be differently expressed in these cells, which lead to the observation that although PC3 have more mesenchymal like characters than DU145, its STIM1 expression is lower than that of DU145. As observed from Fig. 5 and S4, the regulatory effect of ORAI1 on EMT was minor, which was consistent with the observation that PC3 cells show only slight increase of ORAI1 expression level when compared with that of LNCaP and DU145 in Fig.2A.

Studies have shown that STIM1 affects the cell cycle in various cell lines^17^,^21^, and STIM1 knockdown in cervical cancer cells inhibits cell growth by arresting the cells at the S and G2/M phases with increased protein expression level of p21 and decreased expression of Cdc25C and might inhibit Cdk1 activation, leading to cell cycle arrest^12^. However, our study showed that the overexpression of STIM1 slows the growth of DU145 and PC3 cells by reducing the percentage of cells at the G2/M phase and increasing the expression of Myt1, p-WEE1 and p-CDC2. This difference suggests that the effect of STIM1 on cell cycle regulation might be tissue- and cancer type- specific.

Interestingly, we found that STIM1 and ORAI1 promotes the senescence of human prostate cancer cells, as reflected by the aging morphology changes and β-Gal staining in both DU145 and PC3 cells with STIM1 or ORAI1 overexpression and by the overexpression of senescence–related genes, such as *DcR2*. Also of importance, STIM1 down-regulated the expression of anti-apoptotic proteins, including Bcl-2 and XIAP, which in turn might render the prostate cancer cells more susceptible to apoptotic stimuli.

Recent studies have examined the significant role of STIM1 in the metastasis of various types of cancers. It has been suggested that STIM1-mediated SOCE promotes angiogenesis and actomyosin contractility in cervical cancer cells^12^,^49^ and enhances focal adhesion turnover in breast cancer cells^6^. Our data show that STIM1 promotes migration of human prostate cancer cells through its role in regulating EMT via activation of the TGF-β, snail and Wnt/β-catenin signal pathways; thus, this study provides a new understanding of the molecular mechanisms underlying the effect of STIM1 on promoting migration.

Most importantly, we found that both STIM1 and ORAI1 regulate the TME by inhibiting the recruitment of macrophages and by inhibiting formation of TAM. The regulatory effect of SOCE on inflammation has been demonstrated, and an ORAI1 mutation has been reported to be the genetic cause of severe combined immune deficiency (SCID)^4^. In addition, using STIM1 and STIM2 conditional knock-out mice, the function of SOCE in CD8^+^ T cells has been shown to play a vital role in regulating the degranulation of cytotoxic T lymphocytes (CTLs) as well as their expression of the Fas ligand and the production of IFN-γ and TNF-α; thus, SOCE is required to prevent the development of melanoma and colon carcinoma cells^50^ . However, the modulation of the effect of SOCE on the surrounding microenvironment has not been investigated in tumor cells. Our study demonstrated altered protein secretion and mRNA expression levels of multiple cytokine genes, including *IL8*, *CD54*, *CXCL1*, *IFNG*, *IL23A* and *SERPINE1*, all of which are transcriptional target genes of NF-κB^51^,^52^,^53^,^54^,^55^,^56^, in DU145 and/or PC3 cells in which STIM1 is overexpressed. In addition, we also observed decreased mRNA expression of *MIF* and *CXCL1* and overexpression of *IFNG* and *IL23A* in both cells with ORAI1 overexpression. Previous studies revealed the activation of NF-κB by Ca^2+^ influx^57^ or Ca^2+^ oscillation^58^. Here, we observed that the activation of NF-κB was inhibited in prostate cancer cells with increased SOCE activity. Thus, these findings revealed a novel molecular mechanism underlying the effects of both genes on the tumor immuno-microenvironment. Interestingly, although STIM1 has been reported to promote the expression of *VEGFA* in tumor cells, the treatment of macrophages with the culture media from DU145-STIM1-YFP and PC3-STIM1-YFP cells reduced their mRNA expression of *VEGFA* and *MMP9*; combined with the promotion of an unfavorable TME, these factors may therefore compromise the effect of STIM1 on angiogenesis and the metastasis of prostate cancer cells.

The mechanisms underlying the dual effects of STIM1 on prostate cancer are complex, and a recent study showed that some genes or signals, such as TGF-β1, promote cell death and the EMT concurrently in the same types of cell line^59^. Heterogeneity in the cancer cell line or tumor is considered as a key reason; some cells underwent senescence when STIM1 was overexpressed, but those that survived gained the ability to metastasize by undergoing the EMT. It was also suggested that different responses of cells to the same stimuli might also be determined by cell cycle stage and genetic background^60^.

In summary, our study revealed distinct but interrelated effects of SOCE in the progression of human prostate cancer and revealed that STIM1 and ORAI1 are expressed at lower levels in human prostate cancer cells than in normal tissues; in addition, the expression of these molecules is significantly decreased in tumor tissues that exhibit a low differentiation level. On the one hand, STIM1 overexpression promotes the EMT and thereby enhances cell migration; on the other hand, STIM1 overexpression accelerates tumor cell senescence and modulates the tumor immuno-microenvironment. This study thus disclosed novel multiple effects of STIM1 on prostate cancer cells and present new insight into the regulatory mechanism of STIM1 on cell senescence, the EMT and TME; this insight might prove the fundamentality for understanding how different cell fates can be induced by STIM1, even in the same type of prostate cancer.

## Additional Information

**How to cite this article**: Xu, Y. *et al.* STIM1 accelerates cell senescence in a remodeled microenvironment but enhances the epithelial-to-mesenchymal transition in prostate cancer. *Sci. Rep.* **5**, 11754; doi: 10.1038/srep11754 (2015).

## Supplementary Material

Supplementary Information

## Figures and Tables

**Table 1:** The primers used for RT-PCR.

homo *MMP9*	Forward primer: 5′- ATGCGTGGAGAGTCGAAATC -3′
	Backward primer: 5′- TACACGCGAGTGAAGGTGAG -3′
homo *VEGFA*	Forward primer: 5′- TGCTCTACCTCCACCATGCCAAGT -3′
	Backward primer:5′- GCGCAGAGTCTCCTCTTCCTTCAT -3′
homo *IL10*	Forward primer: 5′-GGTTGCCAAGCCTTGTCTGA -3′
	Backward primer:5′- AGGGAGTTCACATGCGCCT -3′
homo *IL6*	Forward primer: 5′- CCTTCGGTCCAGTTGCCTTCT -3′
	Backward primer:5′- CAGTGCCTCTTTGCTGCTTTC -3′
homo *CD163*	Forward primer: 5′- CGAGTTAACGCCAGTAAGG -3′
	Backward primer:5′- GAACATGTCACGCCAGC -3′
homo *IL8*	Forward primer: 5′- ACACTGCGCCAACACAGAAATTA -3′
	Backward primer:5′- TTTGCTTGAAGTTTCACTGGTATC -3′
homo *MIF*	Forward primer: 5′- ACCAGCTCATGGCCTTCG -3′
	Backward primer: 5′- GAGTTGTTCCAGCCCACATT -3′
homo *CD54*	Forward primer: 5′- GCCAGTGGGCAAGAACCT -3′
	Backward primer: 5′- TCAGTGCGGCACGAGAAA -3′
homo *CXCL1*	Forward primer: 5′- CACTGCTGCTCCTGCTCCT -3′
	Backward primer: 5′- GGCTATGACTTCGGTTTGG -3′
homo *IFNG*	Forward primer: 5′- TGTCCAACGCAAAGCAATAC -3′
	Backward primer:5′- TCGACCTCGAAACAGCATCT -3′
homo *IL23A*	Forward primer: 5′- CTGTGGGCCAGCTTCATG -3′
	Backward primer:5′- GGAGGCTGCGAAGGATTT -3′
homo *SERPINE1*	Forward primer: 5′- CAACTTGCTTGGGAAAGGAG -3′
	Backward primer: 5′-GGGCGTGGTGAACTCAGTAT -3′
homo *GAPDH*	Forward primer: 5′- CTCCGGGAAACTGTGGCGTGAT-3′
	Backward primer: 5′-GAGTGGGTGTCGCTGTTGAAGT-3′
