# Supplementary material for: STIM1 accelerates cell senescence in a remodeled microenvironment but enhances the epithelial-to-mesenchymal transition in prostate cancer
Source: Sci Rep. 2015 Aug 10;5:11754. doi: 10.1038/srep11754 (PMC4530453; doi:10.1038/srep11754)
Supplement: Supplementary Information [file srep11754-s1.doc]

**STIM1 accelerates cell senescence in a remodeled microenvironment but enhances the** **epithelial-to-mesenchymal transition in prostate cancer**

**Yingxi Xu^1#^, Shu Zhang^1#^, Haiying Niu^2#^**, **Yujie Ye^1^, Fen Hu^3^, Si Chen^1^,** ^‡^**, Xuefei Li^4^, Xiaohe Luo^1^, Shan Jiang^1^, Yanhua Liu^1^, Yanan Chen^1^, Junying Li^2^, Rong Xiang^1, 5,6^, and Na Li^1, 5,6^***

From ^1^ School of Medicine, Nankai University, 94 Weijin Road, Tianjin, 300071, China

^2^ Department of Obstetrics and Gynecology, the First Central Hospital of Tianjin Medical University, 24 Fukang Road, Tianjin, China, 300192

^3^ School of Physics, Nankai University, 94 Weijin Road, Tianjin, 300071, China

^4^ Beijing Health Vocational College, 94 Nanhengxijie Street, Beijing, China 100053

^5^ Tianjin Key Laboratory of Tumor Microenvironment and Neurovascular Regulation, Tianjin, 300071, China.

^6^ Collaborative Innovation Center for Biotherapy, Nankai University, 94 Weijin Road, Tianjin 300071, China

^^Yingxi Xu, Shu Zhang and Haiying Niu contributed equally to this paper.

**^‡^** Current address: Division of Biochemical Toxicology, National Center for Toxicological Research, FDA, 3900 NCTR Road, Jefferson, AR 72079, USA

*Corresponding author : Dr. Na Li, Tel. (86)-22-23499550; Fax. (86)-22-23502554; Email: [lina08@nankai.edu.cn](mailto:lina08@nankai.edu.cn)

**Supplementary Information**

**Materials and Methods:**

***siRNA transfection***

3 pairs of STIM1 siRNAs and 1 pair of control siRNAs were purchased from Sigma-Aldrich. DU145 or PC3 cells were transfected with 50 nM siRNA-STIM1 mixtures (siSTIM1, ~16.7 nM siRNA for each pair) or 50 nM siRNA-Control (siCtrl) with N-TERTM Nanoparticle siRNA Transfection System (Sigma-Aldrich) following the manufacture’s instruction. The cells were subjected for further experiments after 72 hours’ transfection.

**Table S1. siRNAs used for knocking down *STIM1* and the control.**

| siSTIM1-1 | 5'- GGGAUUUGACCCAUUCCGAdTdT -3' |
| --- | --- |
|  | 5'- UCGGAAUGGGUCAAAUCCCdTdT -3' |
| siSTIM1-2 | 5’- GAGAUUGUGUCUCCCUUGUdTdT -3’ |
|  | 5’- ACAAGGGAGACACAAUCUCdTdT -3’ |
| siSTIM1-3 | 5’- CCUCAAUUACCAUGACCCAdTdT -3’ |
|  | 5’- UGGGUCAUGGUAAUUGAGGdTdT -3’ |
| siCtrl | 5’-UUCUCCGAACGUGUCACGUTT -3’ |
|  | 5’-ACGUGACACGUUCGGAGAATT -3’ |


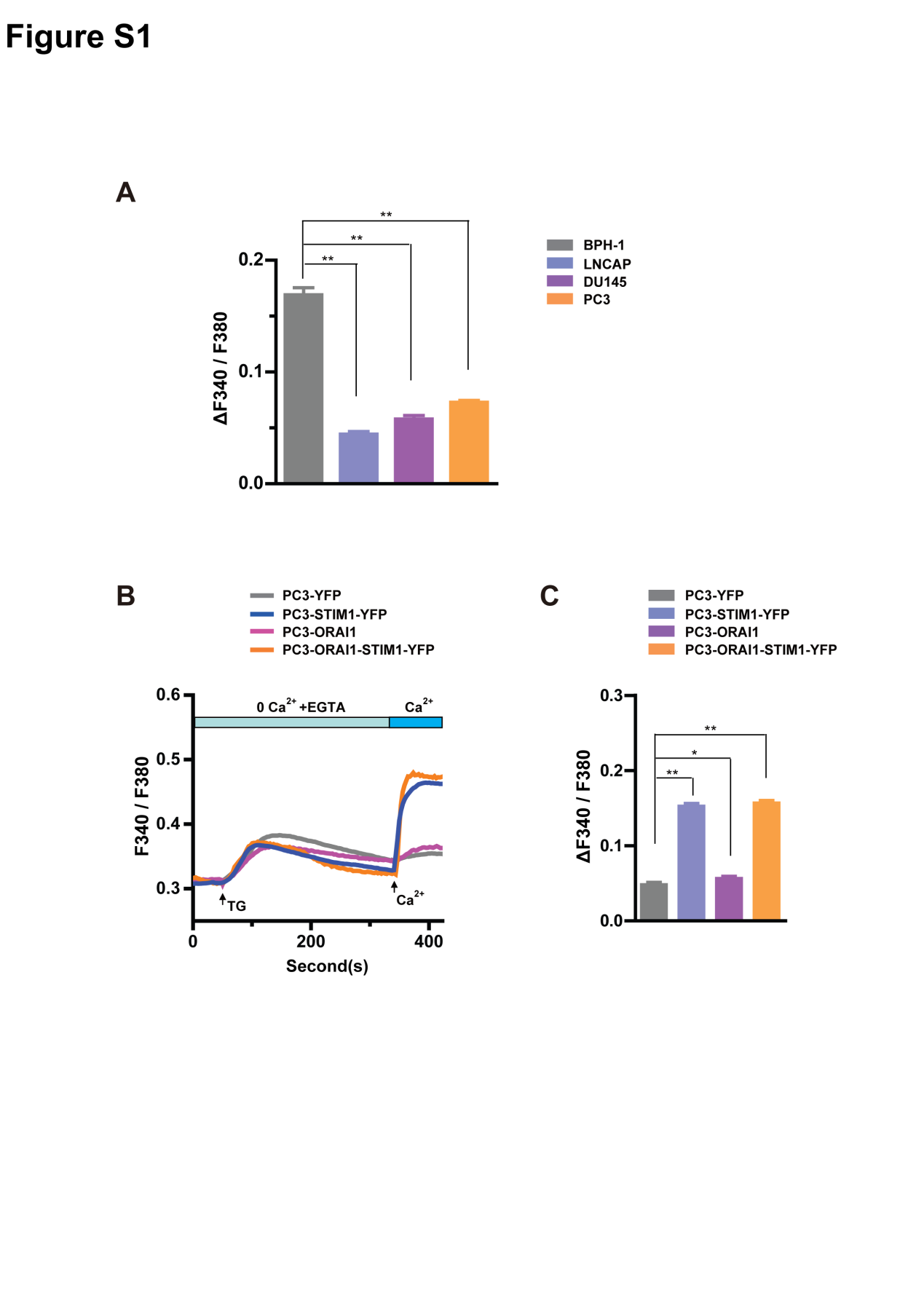


**Figure S1**. Overexpression of STIM1 and/or ORAI1 enhanced the SOCE activity in human prostate cancer cells. **A.** Statistical results of TG induced ER Ca^2+^ store release in human prostate cancer cells, represented as increases in the F_340_/F_380_ ratio over baseline values (ΔF_340_/F_380_) after extracellular TG application. **B.** Comparison of SOCE activity in PC3 cells. **C.** Statistical results of SOCE activities in PC3 cells, represented as increases in the F_340_/F_380_ ratio over baseline values (ΔF_340_/F_380_ ) after extracellular Ca^2+^ application.

**
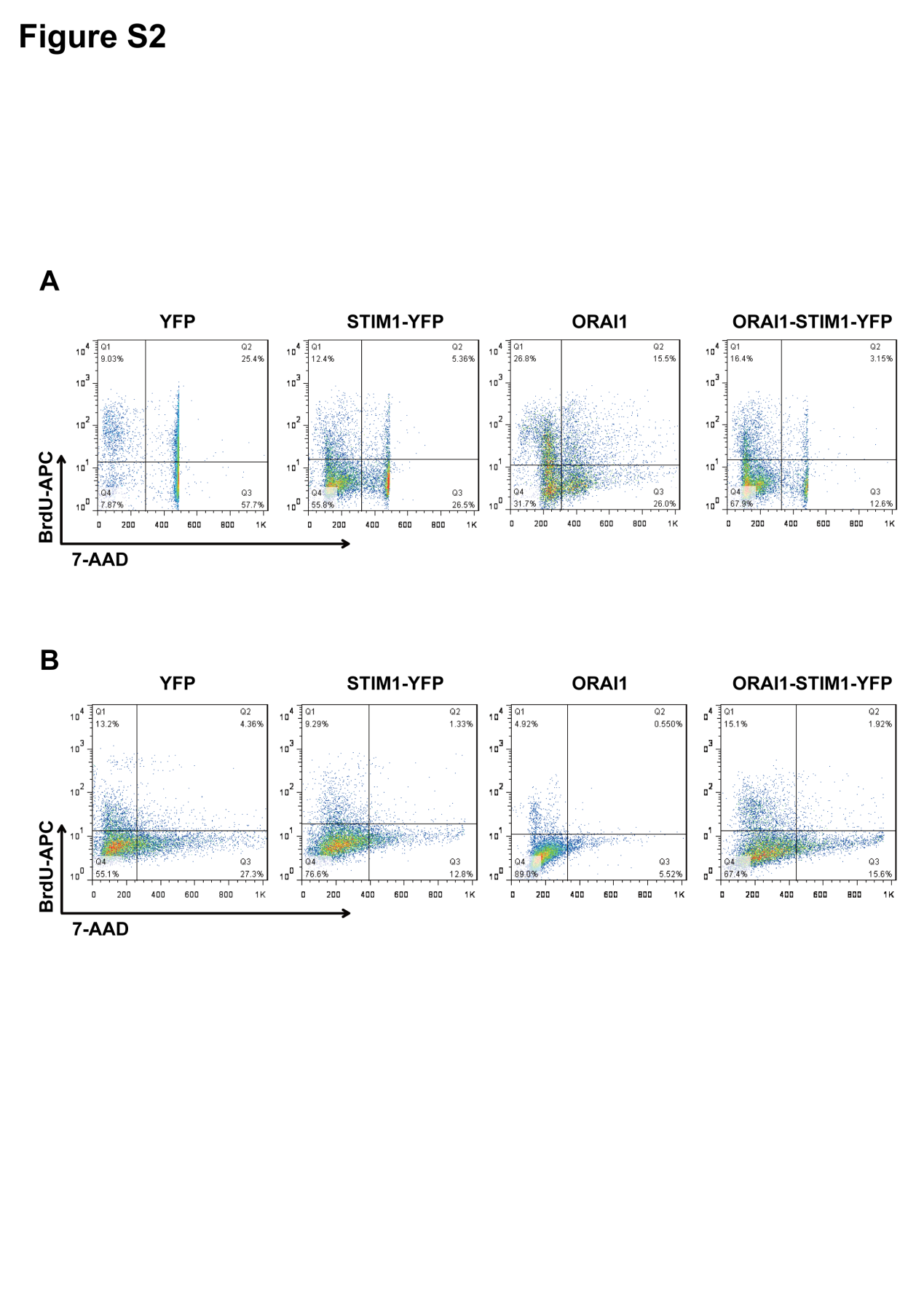
**

**Figure S2**. Representative cell cycle analysis of DU145 (**A**) and PC3 (**B**) cells after staining with 7-Aminoactinomycin D (7-AAD) and BrdU-APC, followed by FACS analysis. Each image represents 1 of 3 FACS analyses. The percentage of cells in the G0/G1, S or G2/M phase was quantified based on the percentage of cells presented in the Q4, Q1+Q2 or Q3 region respectively.


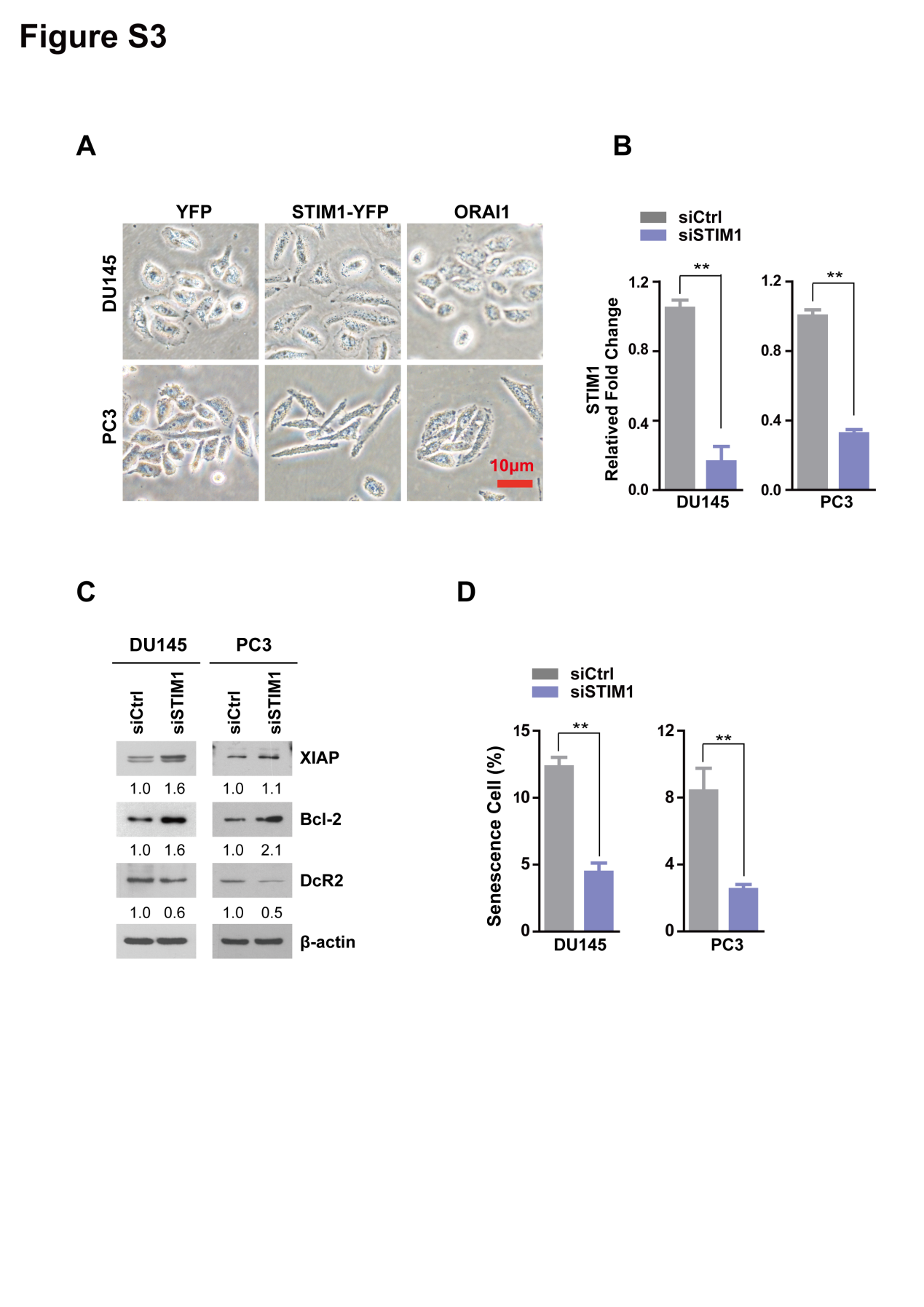


**Figure S3**. STIM1 promotes senescence in human prostate cancer cell lines. **A.** Representative white-field microscopy images of DU145 and PC3 cells overexpressing YFP, STIM-YFP or ORAI1; the images were recorded under a 40× objective. **B.** Real-time RT-PCR results to show the relative mRNA fold change of *STIM1* in DU145 and PC3 cells after transfection with siRNAs targetting STIM1 (siSTIM1) in comparison with the DU145-siCtrl or PC3-siCtrl control (n= 3). **C.** Western blotting results of XIAP, Bcl-2, DcR2 in DU145 and PC3 cells. **D.** Statistical results of senescence cell percentage from the β-Gal staining in DU145 and PC3 cell with *STIM1* down-regulation and the control (n= 3).

**
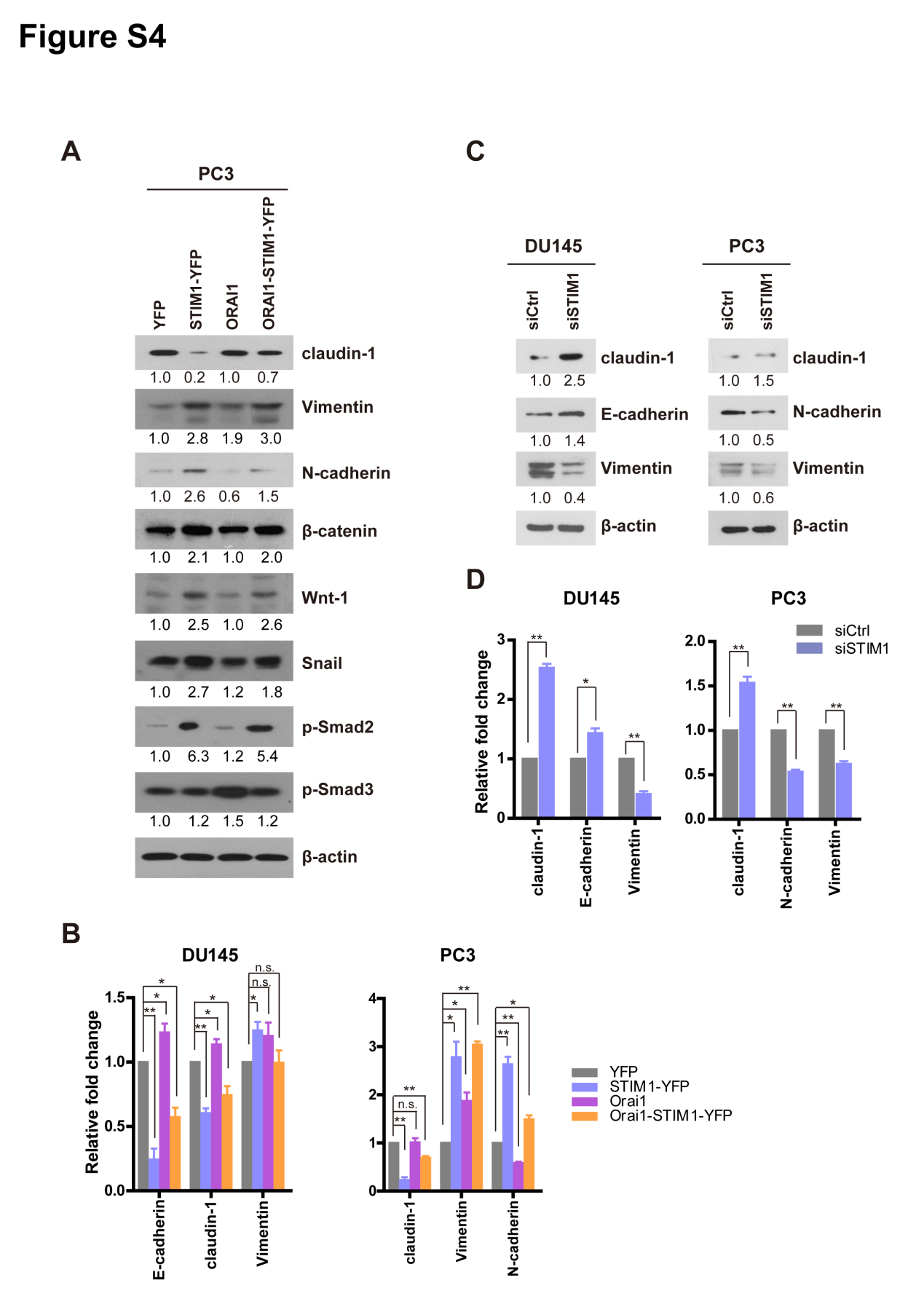
**

**Figure S4**. STIM1 regulates EMT in human prostate cancer cells. **A.**Western blotting analysis of claudin-1,Vimentin, N-cadherin, β-catenin, Wnt-1, Snail, p-Samd2 and p-Smad3 in PC3 cells. **B.** Statistical results of EMT maker protein changes in DU145 and PC3 cells overexpressing STIM-YFP and/or ORAI1 as compared with DU145-YFP or PC3-YFP control (n= 3). **C.** Western blotting results of claudin-1, Vimentn, E-cadherin or N-cadherin in DU145 and PC3 cells with *STIM1* down-regulation and the control. **D.** Statistical results of EMT maker protein change in DU145 and PC3 cells with *STIM1* down-regulation as compared with the control.


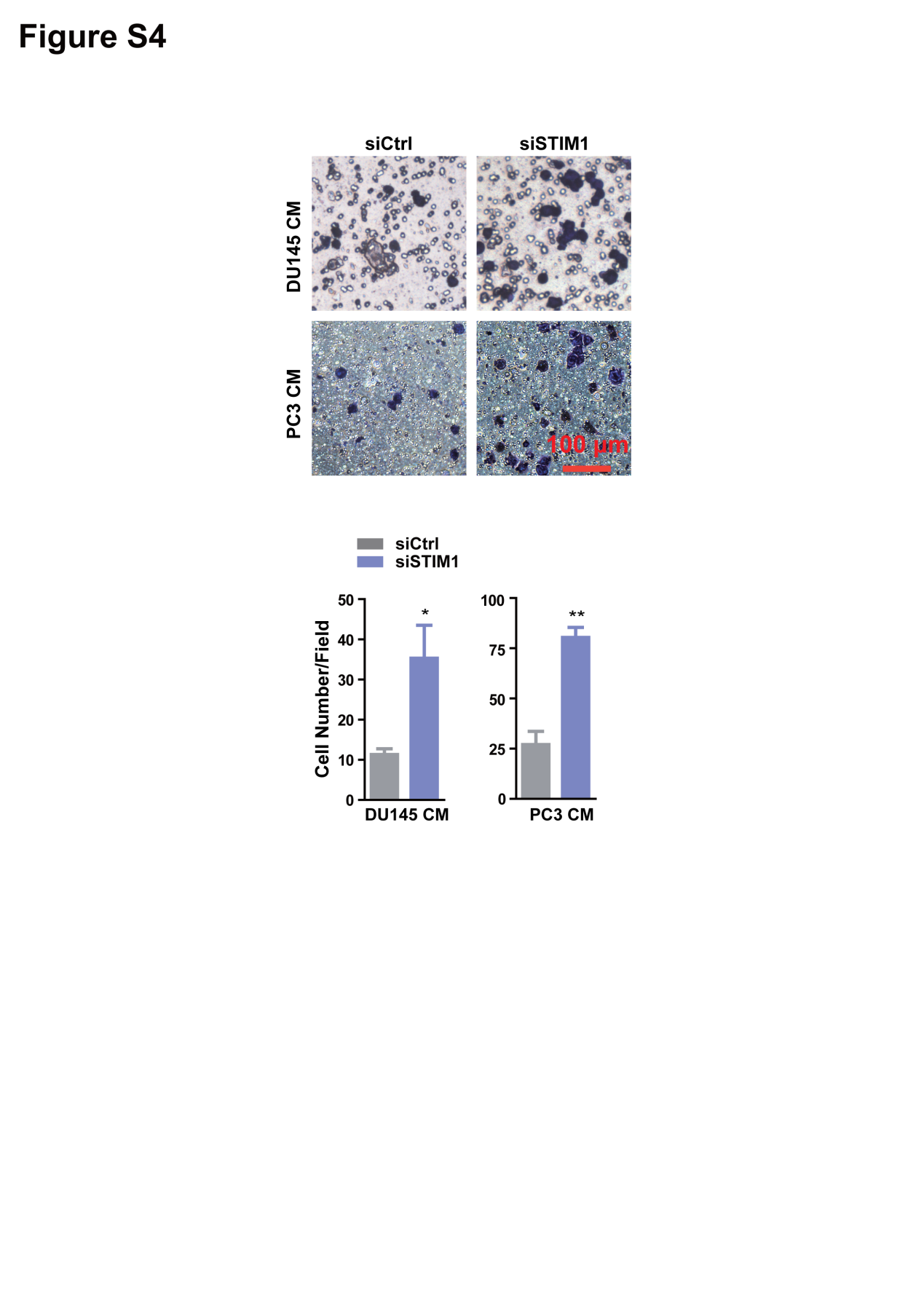


**Figure S5.** STIM1 inhibits the recruitment of macrophages. Upper panel: representative transwell images of U937 that migrated and attached to the bottom of the transwell filter after incubation with the condition medium of DU145 and PC3 cells in lower chamber. Lower panel: statistical results of recruited U973 cell number per image field from transwell assay (n=3).
